# Supplementary material for: Role of Histone Tails and Single Strand DNA Breaks in Nucleosomal Arrest of RNA Polymerase
Source: Int J Mol Sci. 2023 Jan 24;24(3):2295. doi: 10.3390/ijms24032295 (PMC9917218; doi:10.3390/ijms24032295)
Supplement: Supplementary file 1 [file ijms-24-02295-s001.zip › ijms-2165530-supplementary.pdf]

Role of Histone Tails and Single Strand DNA Breaks in Nucleosomal Arrest of RNA Polymerase

Nadezhda S. Gerasimova, Nikolay A. Pestov and Vasily M. Studitsky

SUPPLEMENTARY FIGURES

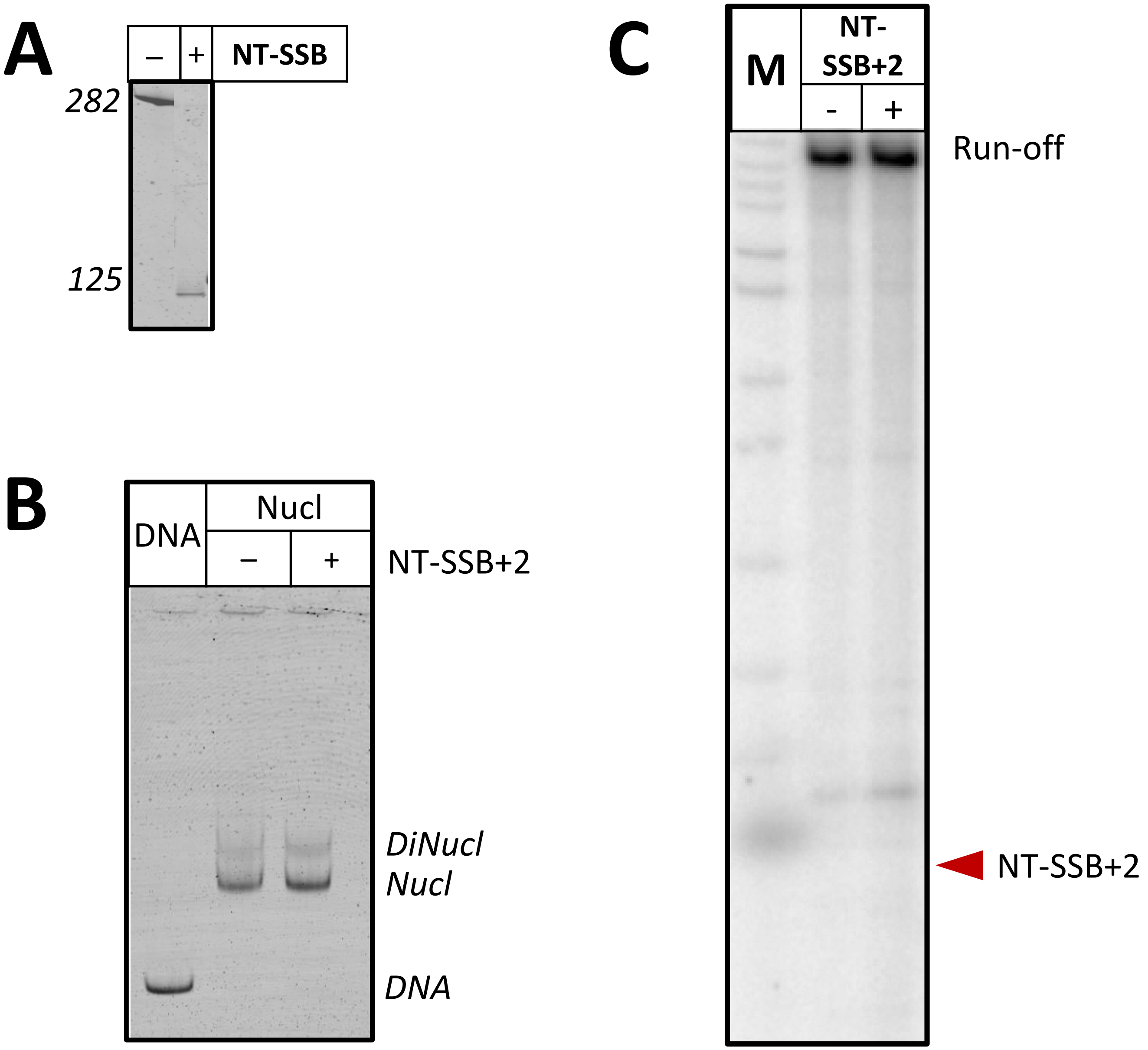

Figure S1. Characterization of the nucleosomes with NT-SSB+2. (**A**) Intact DNA fluorescently labeled at 5’-end of non-transcribed DNA strand was incubated with a nicking endonuclease *Nt.Bsm*A1 to introduce the NT-SSB+2 and analyzed by denaturing PAGE. (**B**) Fluorescently labelled nucleosomes assembled on the intact and NT-SSB-containing DNA were analyzed by non-denaturing PAGE. Other designations as in Fig. 1C. (**C**) Histone-free DNA (intact and with NT-SSB+2) was transcribed for 2 min at 150 mM KCl. Analysis of pulse-labelled RNA in denaturing PAGE. Position of the NT-SSB is shown by the red arrow. M – pBR322 *Msp*I-digest.

A

```
                T7A1up1                                                                                              T7A1up2
5'-P-GATCCCGAAAATTTATCAAAAAGAGTATTGACTTAAAGTCTAACCTATAGGATACTTACA-3'             5'-P-GCCATCGAGAGGGACACGGCGAAAAGCCAACACCGGCACTGG-3'
  3'-CTAGGGCTTTTAAATAGTTTTTCTCATAACTGAATTTCAGATTGGATATC-P-5'                 3'-CTATGAATGTCGGTAGCTCTCCCTGTGCCGCTTTTCGGTTGTGG-P-5'
                T7A1dw1                                                                                              T7A1dw2
```

B

GATCCCGAAAATTTATCAAAAAGAGTATTGACTTAAAGTCTAACCTATAGGATACTTACAGCCATCGAGAGGGACACGGCGAAAAGCCAACACCGG**CACTG**G^GCCAACGCAG
CCCAGTTCGCGCGCCCACCTACCGTGTGAAGTCGTCACTCGGGCTTCTAAGTACGCTTAGGCCACGGTAGAGGGCAATCCAAGGCTAACCACCGTGCATCGATGTTGAAAGAG
GCCCTCCGTCCTGAATTCTTCAAGTCCCTGGGGTACGGATCCGACG

C

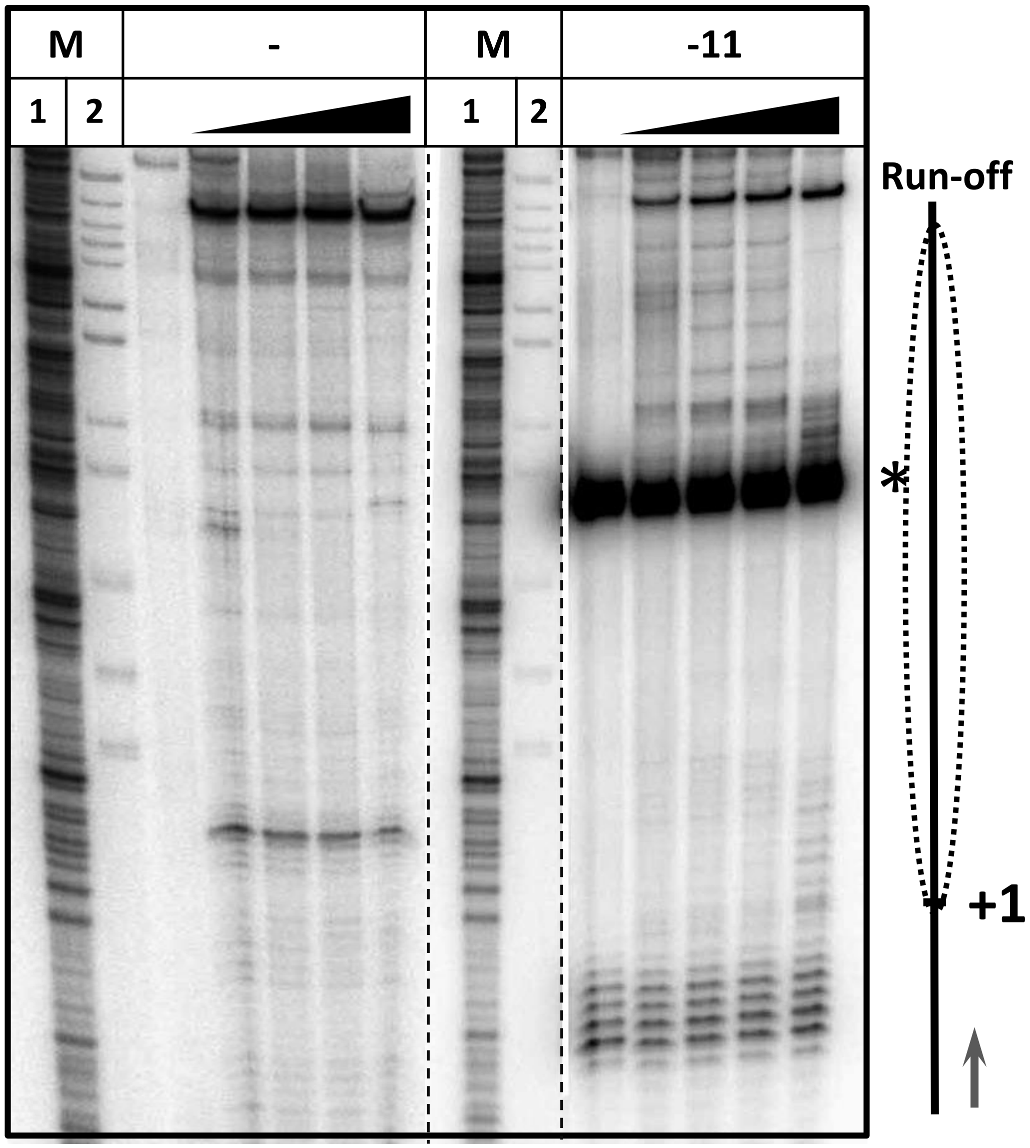

Figure S2. Preparation and transcription of the templates with NT-SSGs. (**A**) Synthetic oligonucleotides composing the promoter part of the fragments are annealed and ligated together to obtain the DNA fragment with a 3' overhang. (**B**) Nucleosomal part is digested using *Tsp*RI and ligated to the promoter part to obtain full-length template with NT-SSG. 603 sequence is underlined. The position of gap (-11) in non-template strand is shown by the sign ^. (**C**) Transcription of histone-free DNA (intact and with NT-SSG-11) by *E. coli* RNAP for 5 min at 40, 150, 300 or 1000 mM KCl. Analysis of the pulse-labeled RNA by denaturing PAGE. M1 – RNA marker. M2 – pBR322 *Msp*I digest. The radioactively labeled fragment of the gap-containing non-template DNA strand (103 nucleotides in length) is shown by the asterisk.

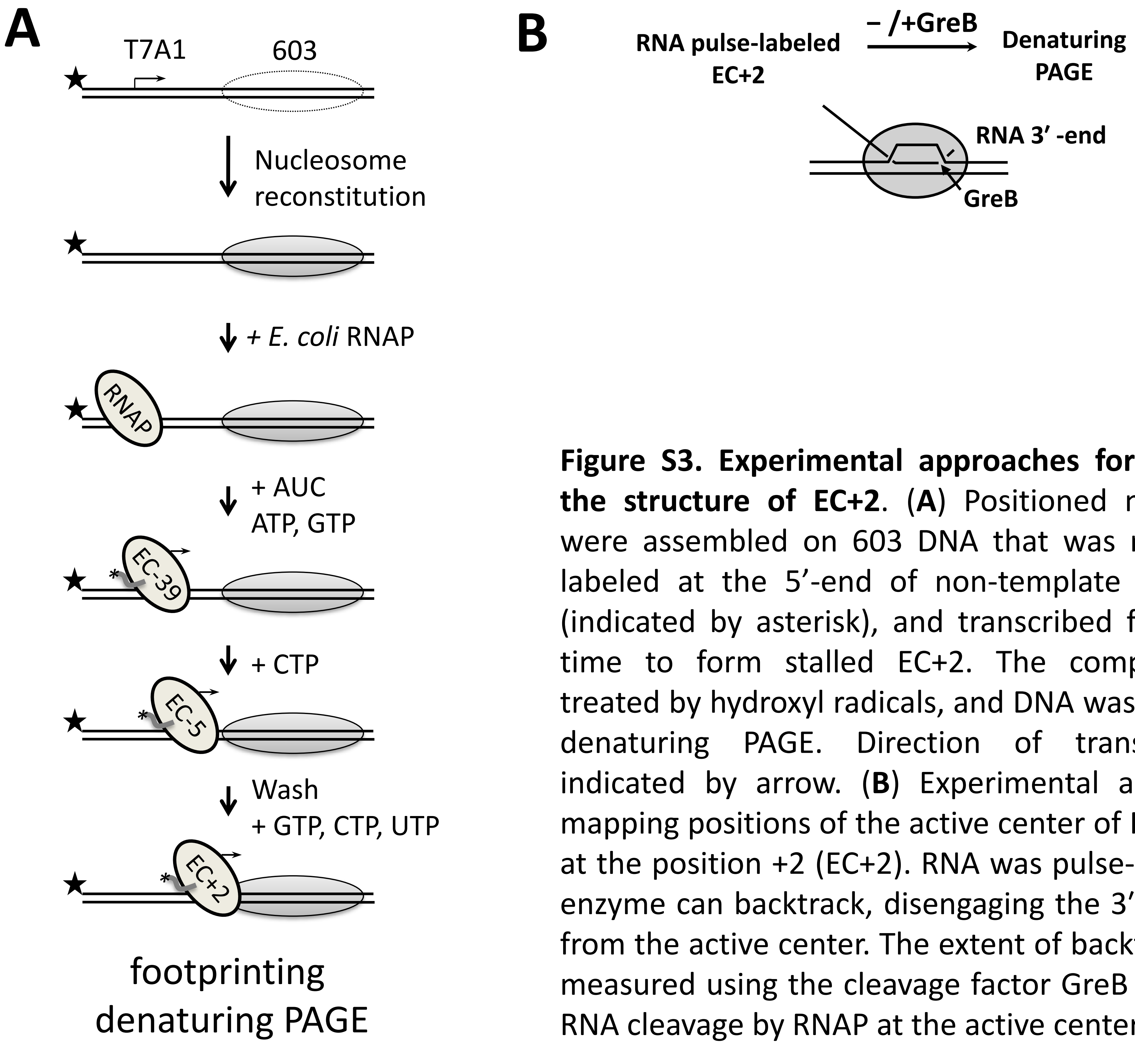

Figure S3. Experimental approaches for analysis of the structure of EC+2. (**A**) Positioned nucleosomes were assembled on 603 DNA that was radioactively labeled at the 5′-end of non-template DNA strand (indicated by asterisk), and transcribed for a limited time to form stalled EC+2. The complexes were treated by hydroxyl radicals, and DNA was analyzed by denaturing PAGE. Direction of transcription is indicated by arrow. (**B**) Experimental approach for mapping positions of the active center of RNAP stalled at the position +2 (EC+2). RNA was pulse-labeled. The enzyme can backtrack, disengaging the 3′ end of RNA from the active center. The extent of backtracking was measured using the cleavage factor GreB to stimulate RNA cleavage by RNAP at the active center.
